# Supplementary material for: Immune-Checkpoint Blockade Therapy in Lymphoma
Source: Int J Mol Sci. 2020 Jul 30;21(15):5456. doi: 10.3390/ijms21155456 (PMC7432396; doi:10.3390/ijms21155456)
Supplement: Supplementary file 1 [file ijms-21-05456-s001.pdf]

Table S1. Results from clinical trials of PD-1 blockade in cHL (including ongoing trials).

Classical Hodgkin Lymphoma					
Trial intervention and setting	Phase	Dosing	n	Response rate	PFS/EFS (IIT)
Nivolumab					
Nivolumab with AVD in untreated patients (CheckMate 205)	phase 2	240mg Q2W	51	ORR 84%, CR 67%	92% at 9months
Nivolumab with AVD followed by IFRT in untreated patients with early-stage unfavorable diease (NIVAHL trial)	phase 2 (randomized)	240mg Q2W	101	concomittant therapy arm; ORR 100%, CR 90%<br>sequential therapy arm; ORR 98% CR 94%	100% at 12 months
Nivolumab with BV in untreated older patients (Age > 60)	phase 2	3mg/kg Q3W	46	ORR 60.9%, CR 45.7%	median 21.8 months
Nivolumab monotherapy in the r/r setting (CheckMate 205)	phase 2	3mg/kg Q2W	243	ORR 66.8%, CR 7%	76.9% at 6 months
Nivolumab with BV in the r/r setting	phase 1/2	3mg/kg Q2W	62	ORR 82%, CR 61%	82% at 21 months
Pembrolizumab					
Pembrolizumab in the r/r setting (KEYNOTE 087)	phase 2	Q3W	210	ORR 71.0%, CR 27.6%	median 13.6 months
Pembrolizumab vs BV in the r/r setting (KEYNOTE 204)	phase 3	Q3W	304	ORR 65.6%, CR 24.5%	median 19.2 months
Trial intervention and setting	Phase	Dosing	status; estimated completion date		ClinicalTrials.gov NCT reference
Nivolumab					
BV+AVD consolidated with nivolumab in untreated patients	phase 2	Q2W	Recruting, August, 2024		NCT03233347
Nivolumab with AVD, ABVD or BV in untreated patients with early-stage disease	phase 2	Q2W	Recruting, August, 2020		NCT03712202
Nivolumab with AVD in untreated patients with advanced-stage disease, compared with BV+AVD	phase 3	Q2W	Recruting, March, 2024		NCT03907488
Nivolumab consolidation after ASCT in high-risk patients	phase 2	Q3W	Recruting, October, 2020		NCT03057795
Nivolumab with ruxolitinib in the r/r setting	phase 1/2	480mg Q4W	Recruting, March, 2024		NCT03681561
Gemcitabine and bendamustine followed by nivolumab in the r/r setting	phase 1/2	Q4W	Recruting, November, 2022		NCT03739619
Nivolumab plus BV with or without Ipilimumab in the r/r setting	phase 1/2	Q3W	Recruting, March, 2025		NCT01896999
Nivolumab with or without BV in r/r setting (Checkmate 812)	phase 3	Q3W	Active, not recruting		NCT03138499
Pembrolizumab					
Pembrolizumab consolidation after ASCT	phase 2	Q3W	Recruting, December, 2022		NCT02362997
Pembrolizumab with AVD in untreated patients	phase 2	Q3W	Active, not recruting		NCT03226249

cHL, classical Hodgkin lymphoma; PFS, progression-free survival; EFS, event-free survival; Q2W, every 2 weeks; Q3W, every 3 weeks; Q4W, every 4 weeks; ORR, overall response rate; CR, complete response; IIT, intension-to-treat; r/r, refractory and relapse; AVD, adriamycin, vinblastine and dacarbazine; ABVD, adriamycin, bleomycin, vinblastine and dacarbazine; IFRT, involved-field radiotherapy; BV, brentuximab vedotin, ASCT, autologous hematopoietic stem cell transplant.

Table S2. Results from clinical trials of PD-1 blockade in DLBCL (including ongoing trials)

Diffuse large B-cell lymphoma					
Trial intervention and setting	Phase	Dosing	n	Response rate	PFS/EFS (IIT)
Nivolumab					
Nivolumab monotherapy in the r/r patients (CheckMate 139)	phase 2	3mg/kg Q2W	121	(ASCT-failed patients) ORR 10%<br>(ASCT ineligible patients) ORR 3%	median 1.9 months<br>median 1.4 months
Nivolumab and ibrutinib in the r/r setting	phase 1	Q2W	45	ORR 36%	NA
Pembrolizumab					
Pembrolizumab monotherapy in the r/r PMBL patients (KEYNOTE 013)	phase 1/2	Q3W	21	ORR 48%, CR 33%	median 10.4 months
Pembrolizumab monotherapy in the r/r PMBL patients (KEYNOTE 170)	phase 2	Q3W	53	ORR 45%, CR 13%	median 5.5 months
Pembrolizumab in patients failing to respond or relapse after CAR-T cell therapy	phase 1/2	Q3W	12	ORR 27%	NA
Durvalumab					
Durvalumab monotherapy in the r/r setting	phase 1/2	Q3W	10	ORR 0%	NA
Durvalumab and rituximab with or without bendamustine in the r/r setting	phase 1/2	Q3W	21	ORR 24%, CR 5%	NA
Durvalumab with R-CHOP or R2-CHOP in untreated high-risk patients	phase 2	Q3W	46	CR 54.1%	NA
Durvalumab with ibrutinib in r/r setting	phase 1/2	Q2W	32	(non-GCB) ORR 38%, CR 31%<br>(GCB) ORR 13%, CR 6%	median 4.1 months<br>median 2.9 months
Atezolizumab					
Atezolizumab with R-CHOP in untreated patients	phase 1/2	Q3W	40	ORR 87.5%, CR 77.5%	74.9% at 24 months
Atezolizumab with obinutuzumab and venetoclax in the r/r setting	phase 2	Q3W	58	ORR 23.6%, CR 18%	NA
Atezolizumab with obinutuzumab and polatuzumab vedotin in the r/r setting	phase 1/2	Q3W	16	ORR 25%, CR 12.5%	NA
Pidilizumab					
Pidilizumab monotherapy after ASCT	phase 2	Q7W	72	ORR 51%, CR 34%	72% at 16 months
Ipilimumab					
Ipilimumab for the r/r setting	phase 1/2	Q4W	18	ORR 11%	NA
Trial intervention and setting	Phase	Dosing	status; estimated completion date		ClinicalTrials.gov NCT reference
Nivolumab					
Nivolumab and Copanlisib in the r/r setting	phase 2	Q2W (cycle1)<br>Q4W (cycle2-)	Recruting, October, 2021		NCT03484819
Nivolumab with duvelisib in the r/r setting	phase 1	Q2W	Recruting, December, 2022		NCT03892044
Nivolumab with or without varilumab in the r/r setting	phase 2	Q2W	Recruting, December, 2020		NCT03038672
Nivolumab and R-CHOP in untreated patients	phase 1/2	Q3W	Recruting, June, 2022		NCT03704714
Nivolumab with DA-EPOCH-R in untreatmed patients	phase 2	Q3W	Recruting, December, 2021		NCT03749018
Nivolumab with lenalidomide in the r/r setting	phase 1/2	Q2W	Recruting, April, 2020		NCT03015896
Nivolumab in patients failing to respond or relapse after CAR-T cell therapy	phase 2	Q4W	Recruting, August, 2022		NCT04205409
Pembrolizumab					
Pembrolizumab with R-CHOP in untreated patients	phase 1	Q3W	Completed, have no result		NCT02541565
Pembrolizumab monotherapy in PD-L1 gene-altered r/r disease	phase 2	Q3W	Recruting, August, 2024		NCT03990961
Pembrolizumab monotherapy after ASCT	phase 2	Q3W	Recruting, December, 2022		NCT02362997
Pembrolizumab with blinatumomab in r/r setting (KEYNOTE 348)	phase 1	Q3W	Recruting, Novenber, 2025		NCT03340766
Pembrolizumab with mogamulizumab in r/r setting	phase 1/2	Q3W	Recruting, July, 2021		NCT03309878
Pembrolizumab with vorinostat in r/r setting	phase 1	Q3W	Recruting, July, 2020		NCT03150329
Pembrolizumab with rituximab in r/r setting	phase 2	Q3W	Recruting, February, 2021		NCT03401853
Pembrolizumab and rituximab with or without lenalidomide in the r/r setting	phase 2	Q3W	Recruting, November, 2021		NCT02446457
Pembrolizumab and radiotherapy in the r/r setting	phase 2	Q3W	Recruting, November, 2020		NCT03210662
Atezolizumab					
Atezolizumab consolidation in high-risk DLBCL	phase 2	Q3W	Recruting, April, 2025		NCT03463057

DLBCL, diffuse large B-cell lymphoma; PFS, progression-free survival; EFS, event-free survival; Q2W, every 2 weeks; Q3W, every 3 weeks; Q4W, every 4 weeks; ORR, overall response rate; CR, complete response; IIT, intension-to-treat; r/r, refractory and relapse; R, rituximab; R2, rituximab and lenalidomide; CHOP, cyclophosphamide, adriamycin, vincristine and prednisolone; DA-EPOCH-R, etoposide, prednisolone, vincristine, cyclophosphamide , adariamycin and rituximab; GCB, germinal center B-cell type; NA, not available; ASCT, autologous hematopoietic stem cell transplant.

Table S3. Results from clinical trials of PD-1 blockade in FL (including ongoing trials)

Follicular Lymphoma					
Trial intervention and setting	Phase	Dosing	n	Response rate	PFS/EFS (IIT)
Nivolumab					
Nivolumab monotherapy in the r/r patients (CheckMate 140)	phase 2	3mg/kg Q2W	92	ORR 4.3%	median 2.2 months
Nivolumab and ibrutinib in the r/r setting	phase 1	Q2W	40	ORR 33%	NA
Durvalumab					
Durvalumab monotherapy in the r/r setting	phase 1/2	Q3W	5	ORR 0%	NA
Durvalumab and rituximab with or without bendamustine in the r/r setting	phase 1/2	Q3W	12	ORR 75%, CR 42%	NA
Durvalumab with ibrutinib in the r/r setting	phase 1/2	Q2W	27	ORR 26%, CR 4%	median 10.2 months
Atezolizumab					
Atezolizumab with obinutuzumab and bendamustine in untreated patients	phase 1/2	Q2W	15	ORR 80%, CR 67%	NA
Atezolizumab with obinutuzumab and lenalidomide in the r/r setting	phase 1/2	Q2W	20	ORR 85%, CR 80%	NA
Atezolizumab with obinutuzumab and venetoclax in the r/r setting	phase 2	Q3W	4	ORR 75%, CR 0%	NA
Atezolizumab with obinutuzumab and polatuzumab vedotin in the r/r setting	phase 1/2	Q3W	7	ORR 57.1%, CR 14.3%	NA
Pidilizumab					
Pidilizumab with rituximab in r/r setting	phase 2	Q4W	32	ORR 66%, CR 52%	median 18.8 months
Trial intervention and setting	Phase	Dosing	status; estimated completion date		ClinicalTrials.gov NCT reference
Nivolumab					
Nivolumab with lenalidomide in the r/r setting	phase 1/2	Q2W	Recruting, April, 2020		NCT03015896
Nivolumab and personalized tumor vaccine in the r/r setting	phase 1	Q2W	Recruting, January, 2028		NCT03121677
Pembrolizumab					
Pembrolizumab with vorinostst in r/r setting	phase 1	Q3W	Recruting, July, 2020		NCT03150329
Pembrolizumab with idelalisib or ibrutinib in r/r setting	phase 2	Q3W	Recruting, January, 2021		NCT02332980
Pembrolizumab with ibrutinib in r/r setting	phase 1	Q3W	Completed, have no result		NCT02950220
Pembrolizumab with rituximab in r/r setting	phase 2	Q3W	Recruting, February, 2021		NCT03401853
Pembrolizumab and rituximab with or without lenalidomide in the r/r setting	phase 2	Q3W	Recruting, November, 2021		NCT02446457
Atezolizumab					
Atezolizumab with obinutuzumab with or without tazemetostat in the r/r setting	phase 1/2	Q3W	Completed, have no result		NCT02220842

FL, follicular lymphoma; PFS, progression-free survival; EFS, event-free survival; Q2W, every 2 weeks; Q3W, every 3 weeks; Q4W, every 4 weeks; ORR, overall response rate; CR, complete response; IIT, intension-to-treat; r/r, refractory and relapse; NA, not available.

Table S4. Results from clinical trials of PD-1 blockade in Virus-associated lymphoma and other types of lymphoma (including ongoing trials)

Virus-associated lymphoma and other types of lymphoma					
Trial intervention and setting	Phase	Dosing	n	Response rate	PFS/EFS (IIT)
Nivolumab					
Nivolumab in r/r PCNSL	phase 1	Q2W	4	ORR 100%, CR 80%	NA
Trial intervention and setting	Phase	Dosing	status; estimated completion date		ClinicalTrials.gov NCT reference
Nivolumab					
Nivolumab with ibrutinib in r/r CNS lymphoma	phase 2	Q2W	Recruting, June, 2023		NCT03770416
Nivolumab consolidation after R-MPV in PCNSL	phase 1	NA	Recruting, May, 2021		NCT04022980
Nivolumab with Pomalidomide in r/r CNS lymphoma	phase 1	Q4W	Recruting, February, 2021		NCT03798314
Nivolumab with GDP and L-apsaraginase in r/r NK/T cell lymphoma	phase 1	240mg, Q2W	Recruting, January, 2022		NCT04230330
Nivolumab with ipilimumab in HIV-associated r/r HL	phase 1/2	Q2W	Recruting, July, 2022		NCT02408861
Pembrolizumab					
Pembrolizumab monotherapy in r/r PCNSL	phase 2	Q3W	Recruting, July, 2022		NCT03255018
Pembrolizumab monotherapy in r/r NK/T cell lyphoma	phase 2	Q3W	Recruting, November, 2021		NCT03728972
Pembrolizumab monotherapy in r/r EBV-associated DLBCL and r/r NK/T cell lyphoma	phase 1/2	Q3W	Recruting, March, 2023		NCT03586024
Pembrolizumab in HIV-related neoplasms	phase 1	Q3W	Recruting, July, 2021		NCT02595866

PCNSL, primary central nervous system lymphoma; CNS, central nervous system; R-MPV, rituximab, methotrexate, procarbazine and vincristine; PFS, progression-free survival; EFS, event-free survival; Q2W, every 2 weeks; Q3W, every 3 weeks; Q4W, every 4 weeks; ORR, overall response rate; CR, complete response; IIT, intension-to-treat; r/r, refractory and relapse; NA, not available.
